# The influence of gender and other patient characteristics on health care-seeking behaviour: a QUALICOPC study

**DOI:** 10.1186/s12875-016-0440-0

**Published:** 2016-03-31

**Authors:** Ashley E. Thompson, Yvonne Anisimowicz, Baukje Miedema, William Hogg, Walter P. Wodchis, Kris Aubrey-Bassler

**Affiliations:** Dalhousie University Family Medicine Teaching Unit, Dr. Everett Chalmers Regional Hospital, 700 Priestman Street, PO Box 9000, Fredericton, NB E3B 5N5 Canada; University of Ottawa, Ottawa, ON Canada; University of Toronto, Toronto, ON Canada; Memorial University, St. John’s, NL Canada

**Keywords:** Health care-seeking behaviour, Primary care, Gender

## Abstract

**Background:**

Canadians’ health care-seeking behaviour for physical and mental health issues was examined using the international Quality and Cost of Primary Care (QUALICOPC) survey that was conducted in 2013 in Canada.

**Method:**

This study used the cross-sectional Patient Experiences Survey collected from 7260 patients in 759 practices across 10 Canadian provinces as part of the QUALICOPC study. A Responsive Care Scale (RCS) was constructed to reflect the degree of health care-seeking behaviour across 11 health conditions. Using several patient characteristics as independent variables, four multiple regression analyses were conducted.

**Results:**

Patients’ self-reports indicated that there were gender differences in health care-seeking behaviour, with women reporting they visited their primary care provider to a greater extent than did men for both physical and mental health concerns. Overall, patients were less likely to seek care for mental health concerns in comparison to physical health concerns. For both women and men, the results of the regressions indicated that age, illness prevention, trust in physicians and chronic conditions were important factors when explaining health care-seeking behaviours for mental health concerns.

**Conclusion:**

This study confirms the gender differences in health care-seeking behaviour advances previous research by exploring in detail the variables predicting differences in health care-seeking behaviour for men and women. The variables were better predictors of health care-seeking behaviour in response to mental health concerns than physical health concerns, likely reflecting greater variation among those seeking mental health care. This study has implications for those working to improve barriers to health care access by identifying those more likely to engage in health care-seeking behaviours and the variables predicting health care-seeking. Consequently, those who are not accessing primary care can be targeted and policies can be developed and put in place to promote their health care-seeking behavior.

## Background

In Canada primary care services provided by physicians are free (paid for through taxation and fees) to all citizens [1]. Except for a few population-based screening programs for certain diseases, patients have to actively seek out primary care services in the community. Primary care can be roughly divided into physical health care, which refers to managing acute or chronic illness and the overall function of the body, and mental health care, which refers to managing conditions such as depression, anxiety and bipolar disorder. Physical and mental health conditions are not mutually exclusive, and there are “multiple associations between mental health and [chronic] physical conditions…” [2]. Due to the structure of the health care system in Canada, patients seeking physical and mental health care usually present initially to the primary care provider [3].

Health care-seeking behaviour, also called health- or help-seeking behaviour interchangeably in the literature, is a complex concept. Cornally et al. [4] use the term help-seeking behavior and defines it as “a problem focused, planned behaviour, involving interpersonal interaction with a selected health-care professional” when seeking help for a health problem. A decade-old report [5] describes two theoretical approaches: the utilization of the formal system or “end point” which is described as health care seeking behavior, and the process concept that includes illness response or health seeking behavior. For the purpose of this paper we will use the term “health care-seeking behavior”.

The literature indicates that patients seek health care more often in response to physical health concerns than for mental health concerns [6]. However, women seek more health care in response to both physical and mental health concerns [7–12]. Even when accounting for increased health care needs unique to women (e.g., pregnancy and related care), research has demonstrated that women visit family physicians more often and report longer consultation times than men [11, 13].

Health care-seeking behavior is influenced by many patient characteristics such as by socio-economic status, gender and age [5]. Other issues such as knowledge of illness prevention and health maintenance, trust in physicians and the presence of chronic conditions have also been demonstrated to impact health care-seeking behavior, including frequency and length of visits, satisfaction and willingness to seek care [10, 11, 14–22]. Adults over 65 years tend to have more consultations with family physicians than younger adults [10, 11, 14]. Older patients also report more positive health care-seeking attitudes regarding mental health concerns than younger patients [7, 23].

Patients with greater health care needs, such as those with chronic conditions and multiple health concerns, use health care to a greater extent than patients with lesser need, and this utilization increases with the number of chronic conditions the patient has [11, 21, 22]. Finally, greater trust in physicians is related to better health outcomes and increased patient satisfaction [16, 19]. In this paper we will examine the predictive utility of a number of factors (gender, age, illness prevention, health maintenance, trust in physicians, chronic conditions) that impact variations in patients’ health care-seeking behaviours for both physical and mental health concerns.

### The current study

The primary objective of the current study is to expand our understanding of health care-seeking behaviour in primary care with regard to mental health concerns. As previous research indicates that gender plays a significant role in patient response to physical health concerns, we expected it also is relevant for mental health concerns.

This research was guided by two research questions: 1) Are there gender differences in the extent to which patients seek health care in response to physical and mental health concerns? 2) What patient characteristics predict the extent to which women and men seek help in response to physical and mental health concerns?

## Methods

The QUALICOPC project is an international study investigating the quality, cost and equity of primary care in countries around the world [24]. Detailed information on the recruitment and administration of the QUALICOPC surveys in Canada can be found elsewhere (see Wong, et al. [25]). Briefly, the project utilized four surveys to examine primary care in Canada; one survey was completed by the physician, one was completed by either the physician or practice staff, and two were completed by patients of the practice. The survey of interest for this study was the *Patient Experiences Survey* (PES) that was completed by patients waiting in primary care provider’s waiting room. Physicians in all 10 Canadian provinces were invited to participate in the Canadian QUALICOPC study [25]. Physicians who agreed to participate were responsible for distributing the PES to their patients, and were compensated with $200 for their efforts; the participating patients were strictly volunteers. The surveys were consistently administered by provincial research teams in each province. Ethics approval was obtained from the research ethics boards at each provincial lead investigator’s institution (see Table 1). For this paper we only used data from the Canadian PES, which consisted of 51 items assessing demographic details and information about participants’ health and experiences with primary care in Canada [24].

**Table 1:** Research Ethics Boards

Province	Institution	Ethics Board
British Columbia	University of British Columbia	Behavioural Research Ethics Board
Alberta	Health Quality Council of Alberta	Community Research Ethics Board of Alberta
Saskatchewan	Health Quality Council of Saskatchewan	University of Saskatchewan Behavioural Research Ethics Board
Manitoba	University of Manitoba	Health Research Ethics Board
Ontario	University of Toronto	Health Sciences Research Ethics Board
	University of Ottawa	Health Sciences and Science Research Ethics Board
Québec	Université de Sherbrooke	Comité institutionnel d’éthique de la recherche avec les êtres humains
New Brunswick/PEI	Horizon Health Network	Research Ethics Board
Nova Scotia	Dalhousie University	Health Sciences Research Ethics Board
Newfoundland & Labrador	Memorial University	Interdisciplinary Committee on Ethics in Human Research

### Measures

Based on the PES survey data, we created a *Responsive Care Scale*, and used single-item questions to assess gender, age, illness prevention, health maintenance, level of trust in physicians, and presence of chronic conditions.

#### Responsive Care Scale (RCS)

An 11-item scale was constructed from the sub-items of one survey question (PES Q25) that measured whether participants *would* visit a family doctor in response to a variety of health concerns. Subsequently, two subscales were constructed from those 11 scale items, separately grouping physical health concerns (6 items; RCS-P) and mental health concerns (5 items; RCS-M). The scale items are presented in Table 1. Items were measured on a 4-point scale (1 = *No*; 2 = *Probably not*; 3 = *Probably yes*; 4 = *Yes*), with higher scores indicating greater inclination to visit a family doctor. There was also a “Don’t know” response option; all of these responses were recoded as missing data. For this study, the overall RCS as well as the two subscales (RCS-P and RCS-M) demonstrated adequate internal consistency (α = .84 for the RCS, α = .71 for the RCS-P, and α = .81 for the RCS-M).

#### Illness prevention

Participants’ confidence in their own ability to prevent illness was assessed using a single question: “How well do you know how to prevent problems with your health?” (PES Q33). Responses were measured on a 5-point scale from 1 (*Hardly confident at all*) to 5 (*Completely*), with higher scores indicating greater confidence in their knowledge. There was also an “I don’t have any health problems” response option; all of these responses were recoded as missing data.

#### Health maintenance

Participants’ confidence in their ability to maintain their health was assessed using a single question: “How confident are you that you can maintain the changes in your health habits, like diet and exercise, even during times of stress?” (PES Q34). Responses were measured on a 5-point scale from 1 (*Hardly confident at all*) to 5 (*Totally confident*), with higher scores indicating greater confidence in their ability.

#### Trust in physicians

Participants’ level of trust in physicians was assessed using a single item: “In general, doctors can be trusted.” (PES Q28-1). Responses were measured on a 4-point scale from 1 (*Strongly disagree*) to 4 (*Strongly agree*), with higher scores indicating greater levels of trust.

#### Chronic conditions

A single dichotomous (yes/no) question assessed whether participants’ had a chronic condition: “Do you have a longstanding disease or condition such as high blood pressure, diabetes, depression, asthma or another longstanding condition?” (PES Q2).

### Analytical strategy

The existence of gender differences in health care-seeking behaviours is well-documented in the literature [5, 7–13]. Therefore, after analyzing the descriptive statistics of the sample, a 2 x 2 mixed-design ANOVA was conducted to investigate gender differences in the extent of participants’ health care-seeking behaviours specifically for physical and mental health concerns. Then, a total of four multiple linear regression analyses were conducted, run separately for men and women to identify what patient characteristics were associated with health care-seeking behaviours for physical and mental health concerns. The dependent variables for the regressions were scores on the RCS-P and RCS-M; the independent variables were age, illness prevention, health maintenance, trust in physicians and chronic conditions. We used an alpha level of .05 for all statistical tests; a Bonferroni correction was applied for the four regressions to avoid inflation of Type I error, setting the significance level at .0125 (*p* = .05/4). All analyses were conducted using IBM SPSS Statistics 21 [26].

## Results

### Sample characteristics

The final sample comprised 7260 patients (4697 women, 2425 men, 138 did not answer) from 759 practices, ranging in age from 19 to 100 years (*M* = 53.33, *SD* = 16.50). Participants were predominantly Canadian-born (83.9 %) and were fluent in French or English (80.2 %). Most had at least one other adult living in their household (75.8 %) and had no children under the age of 18 living in their household (70.5 %). Overall, participants were well-educated, with more than half having completed post-secondary education (58.2 %), and were employed/self-employed (55.1 %) or retired (29.1 %). Most participants were visiting the doctor by appointment (92.3 %), and were there primarily for non-urgent reasons (71.2 %). The most commonly endorsed reasons for the visits were routine medical checks (27.0 %), follow-up appointments (26.5 %), renewing a prescription (22.2 %), and because they were ill or did not feel well (20.4 %).

On average, participants reported moderate health care-seeking-behaviour in response to health concerns (*mean [M]* = 2.90, *standard deviation [SD]* = 0.66). Of the health concerns presented, patients reported the greatest health care-seeking behaviour for blood in the stool (*M =* 3.66, *SD* = 0.74), and the least for relationship problems (*M* = 2.13, *SD* = 1.10). This pattern is the same for both genders. Means and standard deviations for all RCS items are presented in Table 2.

**Table 2:** Means and Standard Deviations of the Responsive Care Scale by Gender

	Total		Women		Men	
Reason	*M*	*SD*	*M*	*SD*	*M*	*SD*
Physical subscale (RCS-P)	3.03	0.67	3.08	0.65	2.96	0.70
Blood in the stool	3.66	0.74	3.70	0.70	3.60	0.79
Stomach pain	3.39	0.92	3.47	0.87	3.24	1.30
Sprained ankle	2.98	1.11	3.07	1.09	2.80	1.14
Removal of a wart	2.93	1.09	3.01	1.07	2.77	1.12
Deteriorated vision	2.64	1.22	2.59	1.22	2.72	1.21
Cut finger that needs to be stitched	2.55	1.14	2.65	1.14	2.54	1.15
Mental subscale (RCS-M)	2.71	0.88	2.81	0.87	2.53	0.87
Anxiety	3.23	1.02	3.36	0.95	2.97	1.08
Sexual problems	2.99	1.10	3.00	1.11	2.97	1.08
Help to quit smoking	2.57	1.30	2.68	1.28	2.37	1.30
Domestic violence	2.51	1.20	2.66	1.20	2.21	1.15
Relationship problems	2.13	1.10	2.23	1.13	1.96	1.02
Total RCS	2.90	0.66	2.96	0.65	2.78	0.68

*Note.* Responsive Care Scale ranges from 1 to 4, with higher values indicating greater inclination to visit a family physician. Italicized items are those conditions for which patients reported greatest and least health care-seeking behaviour

### Gender differences in the extent of patients’ health care-seeking behaviour

A 2 (gender) by 2 (type of health concern) mixed-design ANOVA was conducted to compare the extent of women’s and men’s health care-seeking behaviours in response to physical and mental health concerns. Results revealed a significant between-subject main effect of gender, *F*(1, 6939) = 140.63, *p* < .001, η^2^ = .02. Examination of the means and standard deviations indicated that women (*M* = 2.94, *standard error [SE]* = 0.01) reported they would visit their primary care physician in response to health concerns to a greater extent than did men (*M* = 2.74; *SE* = 0.01). The ANOVA also produced a significant within-subject main effect of type of health concern, *F*(1, 6939) = 1226.51, *p* < .001, η^2^ = .15. The descriptive information indicated that patients reported they would visit a family doctor for physical health concerns (*M* = 3.01, *SE* = 0.08) to a greater extent than for mental health concerns (*M* = 2.67, *SE* = 0.01). However, both of these main effects were qualified by a significant interaction effect between gender and the type of health concern, *F*(1, 6939) = 61.44, *p* < .001, η^2^ = .01. Although univariate follow-up tests suggested that the gender difference for both types of health concerns was significant, the gender difference for the RCS-M [*t*(6950) = −12.43, *p* < .001, *d* = −0.31] was greater than for the RCS-P [*t*(6950) = −7.35, *p* < .001, *d* = −0.18].

### Patient characteristics predicting the extent of health care-seeking behaviour

Because of the gender differences in health care-seeking behaviour, four multiple linear regressions were conducted to examine the predictive utility of age, illness prevention, health maintenance, trust in physicians and chronic conditions separately for women and men. However, a Bonferroni adjustment was used for these analyses to avoid inflation of Type I error; thus, the significance value was set at .0125 (*p* = .05/4). All data was screened according to procedures outlined by Tabachnick and Fidel [27]; all univariate and multivariate regression assumptions were met. In Table 3, we present descriptive information for the independent variables that were used in the regression analyses.

**Table 3:** Descriptive Information for Independent Variables

	Total		Women		Men		
Independent Variable	*M*	*SD*	*M*	*SD*	*M*	*SD*	Range
Age	53.33	16.50	51.48	16.80	56.97	15.23	19–100
Illness prevention	3.69	0.75	3.72	0.75	3.64	0.75	1–5^a^
Health maintenance	3.45	0.95	3.42	0.97	3.50	0.92	1–5^a^
Trust in physicians	3.62	0.51	3.61	0.52	3.64	0.51	1–4^a^
Chronic conditions	0.56	0.50	0.53	0.50	0.62	0.49	0 or 1^b^

^a^ Higher values indicate greater levels of the independent variable

^b^ 0 = no; 1 = yes

The first multiple regression predicted women’s scores on the RCS-M (presented in Table 3). As expected, age, illness prevention, trust in physicians and chronic conditions significantly predicted the extent to which women would visit their primary care physician in response to mental health concerns, *F*(5, 4306) = 23.63, *p* < .001, with the whole model accounting for 2.7 % of the variance. Women who were younger, had greater confidence in their ability to prevent illness, had greater trust in physicians, and had chronic conditions reported that they would visit their primary care physician for mental health concerns to a greater extent than women who were older, had less confidence in their ability to prevent illness, had lower trust in physicians, and no chronic conditions.

The second multiple regression predicted women’s scores on the RCS-P (presented in Table 3). Only trust in physicians significantly predicted the extent to which women would visit their primary care physician in response to physical health concerns, *F*(5, 4349) = 10.15, *p* < .001, with the whole model accounting for 1.0 % of the variance. Women who had greater trust in physicians reported that they would visit their primary care physician for physical health concerns to a greater extent than women who had lower trust in physicians.

The third multiple regression predicted men’s scores on the RCS-M (presented in Table 3). As expected, age, illness prevention, trust in physicians and chronic conditions significantly predicted the extent to which men would visit their primary care physician in response to mental health conditions, *F*(5, 2185) = 16.106, *p* < .001, with the whole model accounting for 3.6 % of the variance. Men who were younger, had greater confidence in their ability to prevent illness, greater trust in physicians and chronic conditions reported that they would visit their primary care physician for mental health concerns to a greater extent than men who were older, had less confidence in their ability to prevent illness, lower trust in physicians and no chronic conditions.

The fourth multiple regression predicted men’s scores on the RCS-P (presented in Table 4). None of the factors significantly predicted the extent to which men would visit their primary care physician in response to physical health concerns, *F*(5, 2201) = 2.387, *p* = .036.

**Table 4:** Summary of Regression Analyses for Predicting the Extent to which Women and Men Seek Primary Care in Response to Mental and Physical Health Concerns

	Women				Men			
	RCS-M*		RCS-P**		RCS-M***		RCS-P****	
Variables	β	*sr*	β	*sr*	β	*sr*	β	*sr*
Age	−.079******	−.074	.025	.023	−.147******	−.141	.051	.049
Illness prevention	.061******	.055	.009	.008	.074*****	.068	.008	.007
Health maintenance	−.020	−.018	.011	.010	−.042	−.039	−.018	−.016
Trust in physicians	.121******	.119	.099******	.098	.089******	.088	.041	.041
Chronic conditions	.079******	.074	.010	.010	.076*****	.073	.019	.018

*Note.* β represents standardized regression coefficient; *sr* represents semi-partial correlation

*N = 4312; **N = 4355; ***N = 2919; ****N = 2207; *****p = .001; ******p < .001

## Discussion

This study examined the characteristics of patients visiting primary care providers in response to a variety of health concerns. It specifically investigated the extent of women’s and men’s health care-seeking behaviours in response to mental and physical health concerns.

Several of the findings confirm what is already known in the literature, such as patients being more inclined to visit their primary care providers in response to physical health concerns than mental health concerns [6]. In addition, we found that women reported that they would visit a family physician in response to both physical and mental health concerns to a greater extent than did men, which is also consistent with other studies [7–12]. Additional examination of facilitators and barriers influencing women’s and men’s health care-seeking behaviours could inform health care initiatives addressing patients’ access to and utilization of primary care.

As expected, we found that age, knowledge of illness prevention, trust in physicians and having chronic conditions significantly predicted health care-seeking behaviour in response to mental health concerns, and this was the case for women and for men. These findings are consistent with previous research [10, 11, 14, 19–22]. However, a new and somewhat surprising finding was that younger patients (women and men) were more willing to seek primary care in response to mental health concerns compared to older patients. This is an encouraging development and we speculate that a number of reasons may be responsible for this shift in health care-seeking behavior. Currently a lot of public health messages, often spearheaded by celebrity Canadians, convey the message that many Canadians suffer from a mental illness and that one should seek help and not be ashamed. In addition, people speak more freely about their struggles with mental health, either in the popular media or via social media and therefore, we speculate, that the stigma around health care seeking behavior for mental health is dismissing.

Most of our variables did not predict health care-seeking behaviour in response to physical health concerns. The sole exception was the finding that women with greater trust in physicians were more inclined to seek health care in response to physical health concerns. Of particular interest is the fact that, contrary to previous research [7, 10, 11, 28], older age did not predict health care-seeking behaviour in response to physical health concerns. Moreover, none of our variables predicted men’s health-care seeking behaviour in response to physical health concerns. It may be that most people would seek health care in response to the particular set of physical health concerns presented in the survey. Finally, knowledge of health maintenance did not predict health care-seeking behaviour for physical or mental health concerns, despite previous research suggesting that increased self-efficacy, in particular for patients with chronic conditions, is associated with improved health outcomes [17].

Although many of our findings were significant due to the large sample, in general the effect sizes were small. Consequently, some of the results may not have meaningful real world implications. Caution must be taken in drawing conclusions until future studies either support or refute them. Nevertheless, we do think that there are clinically significant issues that can be gleaned from the data. First, men are underrepresented in the group of patients seeking primary care in response to both physical and mental health concerns. This has been extensively reported in other literature [7–12], and still remains a concern. Second, it may be suggested that primary care providers should focus on increasing health literacy regarding illness prevention among their patients, so that patients then will be able to better assess when they should seek primary care.

### Limitations and future directions

In the survey, patients were asked to indicate whether they would visit a family doctor for a selection of health concerns. The list of health concerns presented was not exhaustive, but instead covered a range of potential health concerns. We subdivided these concerns into two subscales measuring the extent to which they would seek primary care for physical health concerns and for mental health concerns. As these scales were created from the available survey items, they were not externally published or validated, and studies examining other health concerns may find different results and therefore may be limited in its application. Patients were recruited in family physicians’ offices, therefore orphaned patients’ views are not represented in this sample; hence, our sample is not fully representative of the general Canadian population. In addition, we did not ask patients about their sexual orientation. Research suggests that members of the LGBT community often feel less satisfied with primary care than members of non-LGBT communities [12]. Further, we lacked adequate data regarding participants’ cultural background, education and income, which may also be notable determinants of health care-seeking behaviour [10, 11, 20, 22]. The strength of the study was that the sample was large, representing all 10 Canadian provinces. Thus the trend that younger people are more comfortable seeking care from primary care providers for mental health issues is a robust one.

## Conclusion

Health care-seeking behavior is influenced by different personal characteristics, such as gender, age, knowledge of illness prevention, trust in physicians and having chronic conditions. Men are still underrepresented in primary care; it is difficult to understand why, and this is an ongoing issue. The most important finding—that younger patients, both men and women, are more willing to seek primary care in response to mental health concerns compared to older patients—is encouraging in that it allows problems to be identified and addressed earlier in primary care.

## Consent

Written informed consent was obtained from the patients for the publication of this report and any accompanying images.
